# Indents

**Published:** 1904-07-15

**Authors:** 


### INDENTS.

☞ **Brief Articles of Technical or Scientific Value will receive notice and comment. Contributions invited.**

**Corrosion of Silver Plate.** Silver plates boiled in a solution of cream of tartar and salt will not corrode when placed in the mouth.—*Pacific Gazette.*

**Exposure to Radium Rays.** Mice exposed to radium rays for twenty days lost their hair, which afterward came in bleached. Exposure for a longer period produces muscular paralysis.

**Quick Pickle.** Place work in a glass and nearly cover with hot water. Pour in gradually as much pure sulfuric acid as you have water, and by the time this is done the metal will be clean.—O. Martin, in *Review.*

**To Facilitate Removal of Cap Crowns.** Before setting a cap crown, flow on its interior surface a solution of chloro-percha to make a considerable layer. This will enable one to loosen and more easily remove the crown in case of necessity.—*Summary.*

**Migration of Metals.** An English metallurgist noticed a queer incident, when he placed gold and lead in contact with each other. On later examination he discovered that molecules of gold had traveled as much as two inches into the lead in two years.—*International.*

**Painless Pulp Removal.** Equal parts of chloroform and carbolic acid. Using a French syringe (glass barrel, with glass piston, without needle, simply a canula), pack gutta-percha around the nozzle to prevent escape, and inject, forcing the piston down. The pulp can be immediately twisted out, blanched perfectly white, and insensible to pain.—*International.*

**Removing Rust from Instruments.** Place them over night in a saturated solution of zinc chlorid. The rust will disappear through reduction. Rinse in hot water, place in a hot soap and soda solution and dry. Polish with absolute alcohol and chalk.—*Pacific Medical Record.*

**Soreness of Gingivæ after Crown Setting.** If the soreness is persistent slip over the tooth a ring cut from rubber tubing. Remove carefully the next day and spray the parts with tepid water, and you will in all probability find a minute nodule of cement. Remove and the gum will rapidly heal.—*Review.*

**Glycerite of Tannin for Sensitive Dentin.** Dr. Howland, in the May *International,* recommends as specific for sensitive places at the gingival margins that they be rubbed with a soft stick of wood dipped in a solution of tannin in glycerine. The remedy is very effective and the cure durable.

**Replacing a Broken Facing.** Leave the pins in place. Select a facing of proper size and color, and cut off the pins. Then grind out grooves, to admit the pins left in the bridge, with a suitable disc wheel. Copper and carborundum powder kept wet will do. Undercut grooves and cement to place.—Dr. Bachman, in *Cosmos.*

**To Overcome Shrinkage in Baking Porcelain Inlay.** F. W. Harnden, San Francisco (*Gazette*). To overcome the shrinkage of the first bake from the edges toward the center, make a small hole through the mass just before baking with a pin or needle. The mass will fuse in a ring shape. Have plenty of surplus in the matrix, whether of platinum or gold, and manage to get the corners of this surplus covered with the investing material. This prevents the matrix from changing shape.

**To Remove Inlay Matrix Without Bending.** F. W. Harnden, San Francisco (*Gazette*). A good way to remove a matrix from a tooth cavity is to tie a knot in the end of a thread and lay it in the bottom of the matrix, then cover it with warm wax, which may be pressed perfectly to the cavity walls. When chilled the matrix may be handled with ease.

**Protecting Pyorrhea Pocket.** Dry the root with alcohol and place over the parts bits of Japanese paper saturated with liquid celluloid, so as to form a protecting wall over the gum margins and around the tooth. Tie to place with thread and allow to harden. This forms a shield and protects the granulations which soon form and build up new tissue.—Dr. Younger, in *Summary.*

**To Prevent Drills from Rusting.** Keep the drills in a glass bottle in the bottom of which is placed a pledget of cotton saturated with lysol and the oil used in the Edison battery, the perfumed engine oil will do as well, put the drills, Kerr broaches and similar canal instruments, point downward into the bottle and cork to prevent odors in the office. The instruments should first be cleansed, of course.—L. J. Fernandez, in *Cosmos.*

**Bur for Inlay Cavity Preparation.** Dr. Tompkins, of Utica, N. Y., advocates the use of wheel burs made slightly cone-shape to form cavities with walls diverging at slight obtuse angles to the floor of the cavity. The advantages are that the inlay will adhere better than in a saucer-shaped cavity. The cavity margins are not so attenuated that they are liable to break or discolor. He also suggests an undercut bur, devised to give saucer-shaped cavities a form that will hold cement more securely.—July *Cosmos*.

**Neuralgia, by Alcoholic Injection.** Dr. Tourtelat, of Paris, advocates the injection of alcohol at 140 degrees F. for the treatment of facial neuralgia. The method is to inject the warm alcohol at the point in the mucous membrane of the gum which seems to be the source of the pain. The injection produces a burning sensation and considerable swelling which gradually subsides. The injections may be repeated in a week if necessary. The results may not be permanent, but certainly are helpful as relief measures.—May *Cosmos*.

**To Relieve Tightness of Forcep Joints.** After sterilizing forceps by boiling in various solutions the joints sometimes fail to work easily, because of the accumulation of sodium carbonate, rust, etc. To remove the difficulty, moisten both sides of *open* joint, apply a little loose carborundum powder, No. F or No. 220, and work joint vigorously until it loosens. Then hold under running water and work until gritty feeling leaves the joint, when dry thoroughly and oil. This takes about one minute per pair.—P. W. Smith, Palmyra, N. Y.

**Tooth Powders.**

℞ Prepared Chalk ℥ 3

Fine Pumice

Salol aa ℥ 1½

Add Peppermint Oil to flavor and Carmine to color.

Sig. Use morning and evening with brush and follow with an antiseptic mouth wash. Useful for patients suffering from pyorrhea.

Or ℞ Prepared Chalk
Carbonate of Magnesia aa ℥ 1
Orris Root
Precipitated Sulfur aa ℥ ½
Otter of Roses to flavor

—May *Cosmos*.

**Adrenalin Dangers.** Neugebauer reports that he has seen several cases of localized gangrene following the use of solutions of cocain to which it had been added in the infiltration method of local anesthesia. Elderly persons were especially liable, and he cautions against its use for elderly people. Schucking relates a case where one and three-fourths cubic centimeters of a one to one hundred solution had been injected, and the patient's skin acquired a deep bluish-black color resembling Addison's disease which lasted for half an hour.—*Cosmos* for July, pages 590 and 591.

**Heating Essential Oils in the Teeth.** J. Endelman, in the May *Cosmos*, states that it is not good practice to vaporize applications of the essential oils in root canals or cavities by a warm blast of air, as the oils are volatilized and escape into the air instead of penetrating the dentine as they should. A better plan is to thoroughly desiccate the tooth with alcohol and hot air first, and while the tooth is still warm apply the oil dressing and confine it by a suitable covering, or use pressure and thus force it to permeate the tooth under the most favorable conditions. The idea is logical and is worth remembering.

**Two Methods of Repairing a Crown.** W. Mitchell, London (*Brit. Dent. Jour.*). One is by trephining around the broken pin (when you can not get it out of the root), putting a tube around this pin, fitting the crown on this tube, removing tube and crown for soldering together, and finally cementing into position on the root. The other method is by sawing two slots into the backing as far as the pins, from where the porcelain has broken away then fitting down the tooth, and burnishing over the entire backing and edge of porcelain a thin piece of gold, with two holes punched in to receive the ends of the pins, which are then soldered and cemented, making practically a box crown.

**Preparing Root for Logan Crown.** Anesthetize the labial and lingual portion of the gum and force away with suitable instrument without injuring the gum. Place thin rubber over the root and two adjacent teeth and ligature the stump securely, forcing the ligature well up above the joint line, and carrying the ends of the ligature up and tying around the head, or below the chin as the case may be on upper or lower tooth. Prepare the stump then without injury to the dam or ligature, also the canal. The crown can then be easily fitted and cemented to place with high heat gutta-percha and the dam removed and gum allowed to settle about the tooth, as it will in a short time.—E. R. Griswold, in *Cosmos*.

**Babbitt Metal Dies.** They should be made of copper one part, antimony ten parts, tin eight parts. Ordinary Babbitt metal often contains lead which renders it unfit for dental dies. The counter-dies should be made of lead to which one-sixth of tin has been added to reduce the fusing point so that it can be poured on the die without sticking. When pouring be careful not to pour the counter-die too hot, stir constantly after it is fused until it begins to thicken and pour thickly.

In swaging aluminum plates care must be exercised to prevent tearing because of the inelastic character of the die materials. The counter-dies should be trimmed when necessary to prevent stretching the plate. In gold plates cut at the median line and solder after swaging is completed. This facilitates swaging and makes plate stronger at that point.—Dr. Haskell, in July *Brief*.

**Pressure Anesthesia Needle.** Make for the hypodermic syringe a point with a flaring end, by taking an old reinforced needle and removing the steel needle and soldering to the end of it a cone which can easily be sawed out an old reinforced needle, about an eighth of an inch long, solder this to the end of the point. Three of these points made in varying angles will serve the purpose. Place on an ordinary syringe and it is ready for use. To use cut a small disc of rubber dam or vulcanite that will more than cover the exposure and punch a hole in it with the rubber dam punch, and apply it over the exposure with cavity varnish or chloro-percha to seal it to place. Then fill syringe with cocain solution and apply the bell-shaped point directly over the exposure on the pad of rubber and make gradual and constant pressure. The result will be very satisfactory.—Dr. J. A. Johnson, in *Items*.

**Application of Aluminum Lining.**

Dr. C. W. La Salle, in the July *Cosmos*, gives the following method of applying aluminum bronze to vulcanite plates. Prepare a solvent of carbon disulfid, two parts; naphtha, two parts; chloroform, one part. Pack the case in the usual way having just enough rubber to have the flask come together with slight pressure, and leaving a smooth palatal surface when separated. Take a swab of cotton, and saturate it with the solvent and go over the surface of the rubber, then dip the swab into the aluminum bronze, which may be obtained from the plumber or paint supply house, and apply to the rubber repeatedly until a considerable coat has been applied, keeping the swab wet all the while with the solvent. Apply some dry aluminum to the plaster model and close the flask and vulcanize as usual. After vulcanizing cool the flask thoroughly before opening. This gives a smooth and clean surface to the plate, which is more hygienic than the rubber alone.

**Bridging of Spaces in Soldering.**

Take of fillings of an easy-flowing solder, and fillings of the gold to be soldered, or some higher-fusing metal, about equal parts, also borax and water rubbed up in a mortar or otherwise to make a creamy solution. Mix the fillings with sufficient solution to make a thick paste. Pack the joint to be soldered with this paste and heat till fused. Care should be taken that the entire mass is evenly heated throughout, and of course all the ordinary precautions of having the surfaces bright and clean, etc., must be observed. This method is especially adapted when large spaces are to be bridged, or where it is desirable to add to a cusp or to contour or otherwise change the shape of a piece in any way. Almost any form desired may be obtained, due allowance being made for shrinkage. The particles of high-fusing metal serve as a support to retain the shape of the mass, and the low-fusing solder acts as a cement when fused to unite these particles and bind them to the piece being soldered.—F. W. Stephen, *Summary.*

**Conduct of a Practice.** "I had a new experience to-day," relates a brother practitioner. "About a year ago, the mother of a young miss, whose teeth I had several times refilled with cement preparatory to the time when they would be in proper condition for gold, called at my office and said: 'Doctor, in regard to my daughter's teeth; you have been filling them with cement; Dr. Blank says they should be filled with gold; what do you think?' 'I think they should be filled with cement.' 'But isn't Dr. Blank a good dentist?' 'One of the best in the city.' 'But he says they should be filled with gold.' 'Notwithstanding Dr. Blank's opinion, I still maintain that the teeth are, for the present, better filled with cement.'

"The mother took her daughter to Dr. Blank; to-day the lady returned, but, strange to say, I did not know her; in fact, she found it necessary to introduce herself. She desires to put her daughter in my care again, to do as I please in regard to her teeth. The rest of her family have continued with me. Now, to my old patients I charge the old fee; new patients, new fee. This lady and her daughter come under the new rule and henceforth pay accordingly?"

It is my humble opinion that this gentleman knows how to conduct a dental practice.—W. W. Belcher, in *Office and Laboratory.*

